# Neonatal Leptin Levels Predict the Early Childhood Developmental Assessment Scores of Preterm Infants

**DOI:** 10.3390/nu15081967

**Published:** 2023-04-19

**Authors:** Robert D. Roghair, Tarah T. Colaizy, Baiba Steinbrekera, Réka A. Vass, Erica Hsu, Daniel Dagle, Trassanee Chatmethakul

**Affiliations:** 1 Department of Pediatrics, University of Iowa Stead Family Children’s Hospital, Iowa City, IA 52242, USA; tarah-colaizy@uiowa.edu (T.T.C.); erica-hsu@uiowa.edu (E.H.); daniel-dagle@uiowa.edu (D.D.); 2 Department of Pediatrics, University of South Dakota, Sioux Falls, SD 57105, USA; baiba.steinbrekera@sanfordhealth.org; 3 Department of Obstetrics and Gynecology, Medical School University of Pécs, 7624 Pécs, Hungary; rekaanna.vass@gmail.com; 4 Department of Pediatrics, University of Oklahoma Health Sciences Center, Oklahoma City, OK 73104, USA; trassanee-chatmethakul@ouhsc.edu

**Keywords:** breast milk, cardiovascular, child development, developmental delay, hypertension, neonatology, neurodevelopment, nutrition, outcomes, sexually dimorphic

## Abstract

Preterm infants have low circulating levels of leptin, a key trophic hormone that influences growth and development. While the clinical importance of prematurity-associated leptin deficiency is undefined, recent preclinical and clinical investigations have shown that targeted enteral leptin supplementation can normalize neonatal leptin levels. We tested the hypothesis that, independent of growth velocity, prematurity-related neonatal leptin deficiency predicts adverse cardiovascular and neurodevelopmental outcomes. In a planned 2-year longitudinal follow-up of 83 preterm infants born at 22 to 32 weeks’ gestation, we obtained blood pressures from 58 children and the Ages & Stages Questionnaire (ASQ-3) for 66 children. Based on univariate analysis, blood pressures correlated with gestational age at birth (R = 0.30, *p* < 0.05) and weight gain since discharge (R = 0.34, *p* < 0.01). ASQ-3 scores were significantly higher in female than male children. Utilizing best subset regression with Mallows’ C_p_ as the criterion for model selection, higher systolic blood pressure was predicted by rapid postnatal weight gain, later gestation at delivery and male sex (C_p_ = 3.0, R = 0.48). Lower ASQ-3 was predicted by lower leptin levels at 35 weeks postmenstrual age, earlier gestation at delivery and male sex (C_p_ = 2.9, R = 0.45). Children that had leptin levels above 1500 pg/mL at 35 weeks postmenstrual age had the highest ASQ-3 scores at 2 years. In conclusion, independent of growth velocity, higher leptin levels at 35 weeks’ gestation are associated with better developmental assessment scores in early childhood. While longer-term follow-up of a larger cohort is needed, these findings support investigations that have suggested that targeted neonatal leptin supplementation could improve the neurodevelopmental outcomes of preterm infants.

## 1. Introduction

Infants born before 32 weeks’ gestation are developmentally immature, and they face a gauntlet of obstacles that can impede their achievement of morbidity-free survival. For example, at the time of 2-year follow-up, over 50% of infants born before 27 weeks’ gestational age have moderate to severe neurodevelopmental impairment [1]. With an increasing percentage of premature infants surviving at increasingly earlier gestational ages [1], it is incumbent upon those that assist them on their life journey to fully appreciate their pathway and facilitate its navigation. Among the dramatic changes that occur during the perinatal period is a conversion from transplacental to enteral nutrition during a time when the infant is developmentally unprepared to take control of their growth and nutrition, and maternal breast milk or term formulas are inadequate substitutes for the nourishment that would typically be received prior to birth.

Improved understanding of the nutritional needs of preterm infants, including the need for early protein supplementation, has facilitated body weight increases that often exceed the typical weight gains of the reference fetus [2]. Unfortunately, this overall growth is achieved in the absence of trophic signals, such as leptin, that would have emanated from the placenta to guide not just body size, but also body composition. As a consequence, preterm infants often receive caloric intakes that exceed their capacity for lean body mass accretion, and increased adiposity can be seen at the time of discharge from the neonatal intensive care nursery [3]. Unfortunately, the promotion of growth to improve neurodevelopmental outcomes can come at a cost to long-term cardiometabolic health. In particular, there is a well described direct correlation between infant growth rates and subsequent childhood or adult blood pressures, with stronger correlations seen in men than in women [4,5,6].

In a murine model of neonatal leptin deficiency, we have demonstrated an analogous scenario with improved neurodevelopment but worsening cardiometabolic status following exaggerated caloric intake [7]. We subsequently demonstrated that targeted leptin supplementation without over-nutrition can normalize the neurodevelopment of the leptin-deficient mice without evoking obesity or adult hypertension [8]. In those investigations, male mice were particularly vulnerable to the detrimental cardiovascular and neurobehavioral effects of neonatal leptin deficiency, but if they were provided neonatal leptin replacement, those male mice achieved a reduction in adult blood pressure, reduced sedentary behavior, decreased stress-evoked immobility, and improved brain morphology [8].

The premise that leptin plays a critical role in optimizing perinatal development is supported by its sequential production by the placenta and then the mammary gland [9,10,11]. Regarding the relative importance of leptin production by the placenta rather than the fetus in the sustenance of intrauterine development, placental trophoblastic cells secrete leptin [12], and leptin levels in the umbilical venous blood exiting the placenta exceed the leptin levels in the umbilical arterial blood that is returning to the placenta [13]. Finally, there is a direct correlation between placental weight and cord blood leptin levels [14]. After delivery, while the infant’s adipose tissue remains inadequate to compensate for the loss of leptin delivery by the placenta [14], leptin delivery is possible through colostrum and breast milk [15], and breast-fed infants have higher circulating leptin levels than formula-fed infants [16].

Leptin’s important role in perinatal development and neuromaturation is perhaps best exemplified by patients that have mutations in the leptin gene that have improved brain structure and function after leptin replacement therapy [17,18]. Following the breakthrough discovery that even adults with leptin deficiency can achieve sustained improvements in brain growth with leptin therapy, efforts were made to begin therapy in leptin-deficient children that, due to their ongoing brain growth and maturation, should have an even greater capacity for leptin-evoked brain growth and development [17]. Not surprisingly, case reports soon showed that leptin therapy improved the metabolic status and neurocognitive outcomes of children with monogenic leptin deficiency [18,19].

Far beyond the number of individuals with autosomal recessive leptin deficiency, a growing population of infants have environmentally induced leptin deficiency as a consequence of preterm delivery. As others have similarly shown [20], we recently described pervasive and profound neonatal leptin deficiency in infants born at 22 to 32 weeks’ gestation [21]. In that investigation, neonatal leptin levels fell precipitously from a median of 1303 pg/mL to a median of 242 pg/mL within days of delivery, and remained over 10-fold below the levels seen in cord blood samples obtained at the corresponding postmenstrual ages, with the lowest levels seen in male infants [21]. Consistent with those results, Ertl and colleagues also demonstrated lower leptin levels in preterm male infants, and went on to correlate those leptin levels with the newborn’s testosterone level, suggesting that testosterone may suppress leptin synthesis by the placenta or fetal adipose tissue [20].

The etiology of the leptin deficit of prematurity is likely multifactorial with important considerations including the loss of transplacental leptin delivery at a time when adipose tissue reserves are underdeveloped, and the absence of clinically significant amounts of leptin in formula or Holder pasteurized donor milk [22]. The lower levels of leptin in donor milk compared to a preterm infant mother’s own milk is likewise multifactorial, with important contribution from the deficit in leptin within the milk from the mothers of term versus preterm infants and the near complete removal of leptin from the donor milk following the pasteurization process [22]. Related to the detrimental effects of Holder pasteurization, we recently reported that plasma leptin levels among preterm infants at 34 to 36 weeks postmenstrual age (PMA) significantly increased from 989 pg/mL during receipt of donor breast milk to 1774 pg/mL during receipt of maternal breast milk [23].

Prematurity is a known risk factor for future hypertension and neurodevelopmental impairment [24,25], but it is unclear whether prematurity-related leptin deficiency plays a role in the short and long-term complications of premature birth. We leveraged our prospective cohort of preterm infants to test the hypothesis that, independent of growth velocity, prematurity-related neonatal leptin deficiency predicts sex-specific adverse cardiovascular and neurodevelopmental outcomes, as routinely assessed during 2-year follow-up examination. We found that leptin levels at 34 weeks’ gestation did not predict blood pressures, but they did correlate with neurodevelopmental scores. This is the first study that we are aware of that demonstrates an association between late gestation leptin levels and the short-term developmental outcomes of very preterm infants.

## 2. Materials and Methods

Infants born between 22 0/7 and 32 6/7 weeks’ gestation without congenital anomalies admitted to the University of Iowa Stead Family Children’s Hospital were prospectively enrolled in an observational longitudinal study to describe neonatal leptin levels and their associations with the typical morbidities of prematurity [21]. Infants with major congenital anomalies were excluded. As previously described, the newborn infant’s blood leptin levels were measured by weekly collection of 200 microliters of blood into EDTA tubes during previously scheduled lab draws. Plasma was isolated within 4 h of collection and stored at −80 degrees Celsius prior to analysis. The plasma samples were analyzed in duplicate using a customized magnetic bead assay (Millipore Sigma, Burlington, MA, USA) on BioPlex 200 with BioPlex manager 6.1 software (Bio-Rad, Hercules, CA, USA). Based on our data showing that enteral feedings influence plasma leptin levels at 34–36-week PMA [23], the plasma samples obtained at 35-week PMA were used to test our overarching hypothesis that modifiable neonatal leptin levels can predict early childhood outcomes. Infant gestational age at birth, birth weight, age at discharge, and discharge weight were extracted from the electronic medical record (Epic, Verona, WI, USA) and stored in REDCap version 8.3.2 (Vanderbilt, Nashville, TN, USA).

With approval from the University of Iowa Institutional Review Board (IRB# 201510835), we attempted to contact a parent or guardian of the 145 infants included in our longitudinal follow-up study two years following their child’s birth. Fifty-eight families were lost to follow-up, and 4 refused extraction of outpatient data. We obtained consent to extract data obtained during the 2-year follow-up assessment from 83 families. Childhood demographic and clinical data were then collected from the electronic medical record (Epic, Verona, WI, USA), including current weight, blood pressure, and Ages & Stages Questionnaire (ASQ-3). The ASQ-3 is a widely used and validated parent-completed questionnaire that screens for communication, gross motor, fine motor, problem solving, and personal-social delays [26,27].

Student’s two-tailed t-tests were used for comparison of blood pressures or ASQ-3 scores between male and female children. Simple linear regression was used to determine the relationship of blood pressures or ASQ-3 scores with each of the predictor variables of interest, including gestational age at birth, birth weight, leptin level at 35-week PMA, current weight, and weight gain since discharge. To identify the best predictive model for each of the dependent variables, best subset regression was performed with Mallows’ C_p_ as the criterion for model selection with a variance inflation factor (VIF) below 3 used to define acceptable collinearity, and a target C_p_ that approaches the number of included predictor variables used to minimize the likelihood of significant bias in the chosen model. SigmaPlot 14 (Systat Software Inc., San Jose, CA, USA) was used for statistical analysis, and statistical significance was defined by *p* < 0.05.

## 3. Results

Among the 83 participants, 45% (N = 37) were female and 55% (N = 46) were male. Sex-specific blood pressures and ASQ-3 scores are provided in Table 1. Overall, based on SBP above the 95th percentile, 15 children (26%) were hypertensive and another 5 children (9%) with SBP between the 90th and 95th percentiles were pre-hypertensive [28].

**Figure 1:**
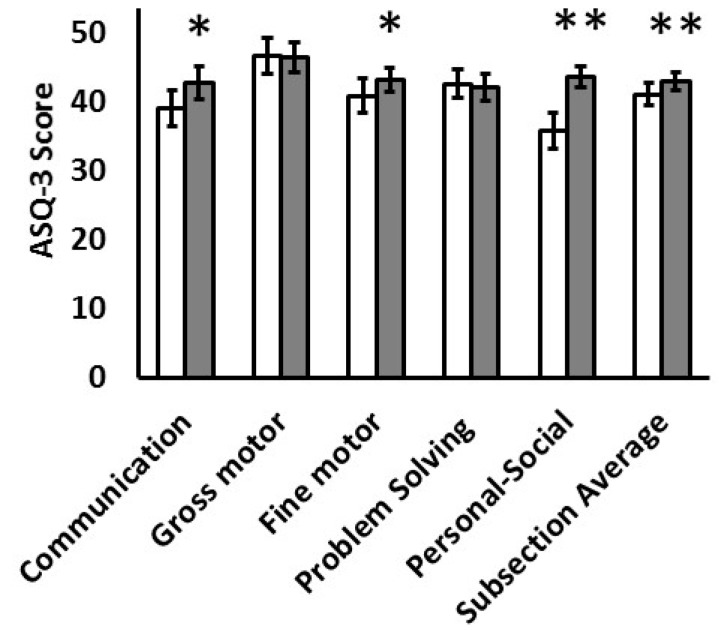
ASQ-3 subsection scores are contrasted for male children (white bars, N = 34) and female children (black bars, N = 32) across the 5 domains of communication, gross motor, fine motor, problem solving and personal-social development. The average score across the 5 subsections is also provided. * *p* < 0.05 or ** *p* < 0.01.

ASQ-3 subset scores were significantly higher for females than males in the domains of communication, fine motor and personal-social (Figure 1). Overall, at least one domain score was below the cutoff for developmental assessment referral in 7 (22%) females and 19 (56%) males (Chi-Square *p* < 0.01).

**Figure 2:**
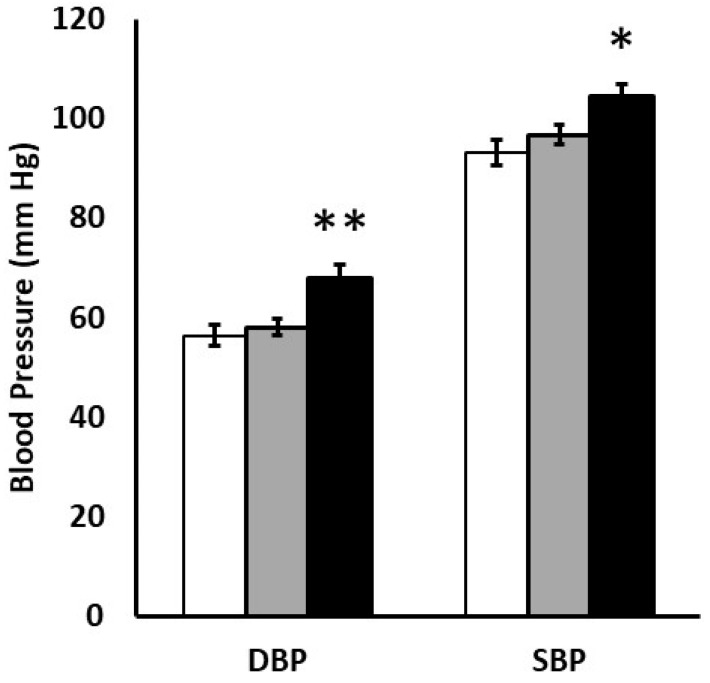
Diastolic blood pressure (DBP) and systolic blood pressure (SBP) are contrasted for children with slow weight gain velocity (<10 g/d, open bars, N = 18), typical weight gain velocity (10–15 g/d, gray bars, N = 30) or rapid weight gain velocity (>15 g/d, black bars, N = 10). * *p* < 0.05 or ** *p* < 0.01 versus 10–15 g/d cohort.

On univariate analysis (Table 2), SBP correlated with gestational age at birth, and DBP correlated with follow-up weight. SBP and DBP both correlated with birth weight and weight gain since discharge, but not 35-week PMA leptin levels. Overall, children with weight gains exceeding 15 g/d had significantly higher outpatient SBP and DBP than peers with typical weight gain velocity of 10–15 g/d (Figure 2). In unadjusted analysis, ASQ-3 was not significantly associated with gestational age, birth weight, leptin levels or weight gain since discharge (Table 2). 

**Figure 3:**
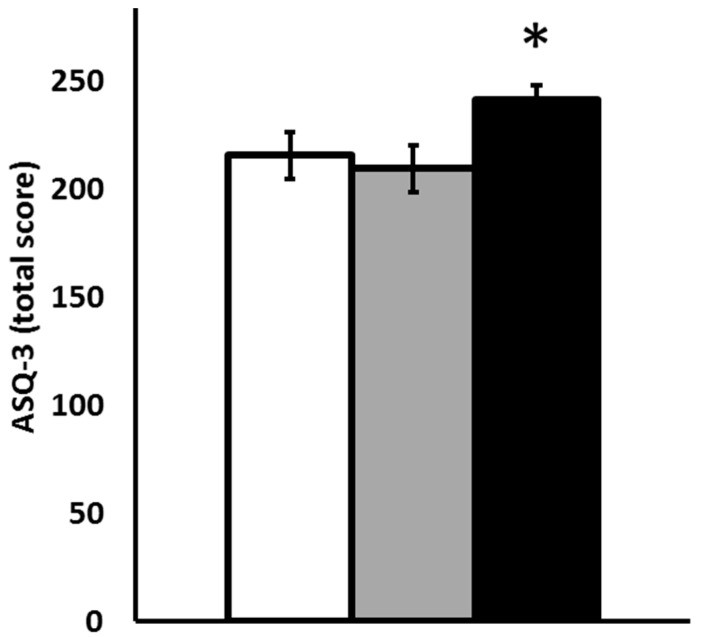
ASQ-3 are contrasted for children with leptin levels at 35-week PMA <500 pg/mL (open bars, N = 25), 500–1500 pg/mL (gray bars, N = 20) or >1500 pg/mL (black bars, N = 11). * *p* < 0.05 versus cohort with levels of 500–1500 pg/mL by Student’s *t*-test.

By best subset regression using Mallows’ C_p_ as the criteria for model selection, higher SBP was predicted by male sex, later gestational age at delivery, and more rapid postnatal weight gain (C_p_ = 3.0, R = 0.48, VIF = 1.02 to 1.04). Higher DBP was predicted by higher follow-up weight and faster postnatal weight gain (C_p_ = 4.1, R = 0.47, VIF = 1.00). Lower ASQ-3 was predicted by male sex, earlier gestational age at delivery, and lower leptin levels at 35-week PMA (C_p_ = 2.9, R = 0.45, VIF = 1.05 to 1.09). The relationship between leptin levels and ASQ-3 score is shown in Figure 3. Infants with leptin levels over 1500 pg/mL (reflective of the leptin levels that were detected among maternal breast milk-fed infants in our prior investigation [23]) had significantly better scores on ASQ-3 assessment than a reference cohort with leptin levels at 35-week PMA that were 500–1500 pg/mL (reflective of the leptin levels we detected among donor milk-fed infants in our prior investigation [23]). All ASQ-3 subsection scores were highest in the infants with 35-week PMA leptin levels over 1500 pg/mL, and statistical significance (*p* = 0.01) was seen for the gross motor development of infants with 35-week PMA leptin levels that were >1500 pg/mL having a median (IQR) of 60 (55–60) versus infants with 35-week PMA leptin levels that were 500–1500 pg/mL having a median (IQR) of 42.5 (25–55).

## 4. Discussion

In the context of profound cardiometabolic and neurodevelopmental abnormalities among patients with congenital leptin deficiency [29,30,31,32], ongoing investigations have highlighted converging lines of evidence in support of the hypothesis that the precipitous and prolonged leptin deficiency seen in premature infants likewise contributes to an increased risk of hypertension and developmental impairment [21,24,25]. From a basic science perspective, hypothalamic and cortical development is positively impacted by ambient leptin levels during the later stages of human embryogenesis [33,34]. When evaluating animal models, it is important to consider the differing developmental trajectory between species. Specifically, while human and non-human primates are altricial species, rodents born at term gestation have more profound neurodevelopmental immaturity with brain development most analogous to 34-week preterm infants [35]. As a result, we have joined others in modeling the neonatal leptin deficiency of preterm infants in neonatal rodents, and demonstrated cardiovascular and neurodevelopmental impairments that could be rescued by neonatal leptin replacement [8,36,37]. Emphasizing the commonality between mammalian models, leptin has been shown to have neuroprotective effects in both rodents and non-human primates [38,39]. Against that backdrop, we returned to the bedside to obtain novel longitudinal data to further interrogate the potential correlation between the leptin deficit of prematurity and the adverse outcomes often seen in preterm infants. We specifically sought to expand on the existing literature by evaluating the association of modifiable neonatal leptin deficiency with short-term neurodevelopmental and cardiovascular outcomes within the context of known risk factors for those adverse outcomes. While we did not identify a correlation between leptin and outpatient blood pressures at the time of 2-year follow-up, leptin levels obtained at 35-week PMA did predict short-term neurodevelopmental outcomes, as reflected by standard ASQ-3 screening.

Preterm delivery is an established risk factor for adult hypertension [24]. Consistent with our results, Duncan and colleagues previously demonstrated an increased incidence of hypertension (15%) and prehypertension (9%) in very low birth weight infants at 2 years of age [40]. Interestingly, those investigators did not see an association between the degree of prematurity and follow-up blood pressures, and we observed a direct correlation, rather than an inverse relationship, between gestational age at delivery and blood pressure. The presence of hypertension or pre-hypertension in more than 30% of our cohort and the exclusion of infants born after 32 weeks’ gestation, which would likely have a lower incidence of hypertension, likely contributed to discrepancy in the direction of the correlation we identified between gestational age and blood pressure versus the existing literature [24].

Beyond the gestational age at delivery, accelerated growth velocity after delivery is an established and synergistic risk factor for subsequent hypertension [4,5,6]. In the prospective Project Viva cohort, Belfort and colleagues observed an independent association for weight-for-length z-scores through 6 months and systolic blood pressures at 3 years with SBP 1 mm Hg higher for each z-score increment [4]. Similarly, Law and colleagues identified a 1.6 mm Hg increase in male and female adult SBP for each standard deviation score increase in early childhood weight gain, and as seen in our investigations, the effect of postnatal weight gain surpassed the effect of birth weight on blood pressure [6]. The direct relationship that we observed between weight gain and blood pressure is also consistent with results of others that have shown detrimental cardiometabolic parameters with early adiposity rebound [41,42]. We did not assess body composition or outpatient leptin levels, but other studies have suggested that leptin levels in early childhood could potentially predict later cardiometabolic risk [43]. In a recent investigation, leptin levels were higher in the breast milk of mothers of preterm than term infants, again suggesting an evolutionary role for leptin in infant development, but the differential leptin levels present in breast milk did not negatively impact the neonatal growth of the preterm or term infants [44]. Interestingly, Project Viva investigators obtained longitudinal leptin levels throughout childhood and showed that children with gradually increasing leptin levels had greater adiposity, as would be expected given the production of leptin by adipose tissue, but they also had lower SBP than those that lacked increasing childhood leptin [45].

A significant proportion of our cohort had screening results suggestive of developmental delay, and this was far more common among males. While male infants are known to have increased risk of prematurity-related developmental delay [46], the etiology of that sexual dimorphism has not been elucidated. Among the neonatal parameters associated with poor motor outcomes, increasing duration of postnatal steroid treatment was the major risk factor [46]. While glucocorticoids have myriad effects that could contribute to that association, it is concerning that dexamethasone can directly suppresses central leptin receptor signaling, with both in vitro and in vivo experiments identifying glucocorticoid-induced inhibition of leptin-mediated activation of the Janus kinase-signal transducer and activator of transcription downstream pathway [47]. Further, clinical investigations have consistently demonstrated higher leptin levels in female than male infants [21,48], and that sexual dimorphism may likewise be driven by differences in steroid exposure, with testosterone associated with reduced leptin synthesis throughout the lifespan [20,49].

Preclinical studies have shown improved neuroanatomic and behavioral outcomes with leptin supplementation [8,33,37]. In our murine model of neonatal leptin deficiency, neonatal leptin replacement improved locomotor activity, the response to stressful environments and brain structure by magnetic resonance imaging, particularly among male mice [8]. In a follow-up investigation, we assessed murine behaviors that partially model some of the features of human autism spectrum disorder or attention deficit hyperactivity disorder, and again noted that neonatal leptin therapy normalized the social interaction and visuospatial learning of leptin deficient mice [37]. Similarly, when newborn rats are separated from their mothers, they develop sex-specific leptin deficiency and behavioral alterations that can be improved by neonatal leptin replacement [36].

Independent of overall protein intake, breast milk leptin intake on postnatal days 7 to 28 has been correlated with improved weight gain and head circumference of preterm infants at 36-week PMA [50]. In addition to higher ASQ-3 scores in children with the highest leptin levels, we found that leptin levels contribute to the use of gestational age and sex in the prediction of ASQ-3 scores. Additional support for an important role for a relationship between milder forms of leptin deficiency and neurodevelopment was recently provided by a study of term infants in rural China that showed cord blood leptin levels positively predicted the infants’ scores on the Bayley Scales of Infant and Toddler Development-Third Edition [51]. Given the very low leptin levels seen in our cohort and the high frequency of abnormal screening scores, further research is needed to determine if leptin supplementation or optimized breast milk processing methods could be leveraged to increase leptin delivery and subsequent outcomes. While Holder pasteurization consistently reduces the leptin content of breast milk to nearly undetectable levels [22,52,53], high-temperature short-time treatments have been shown to yield leptin retention rates between 34 and 68% [52]. Alternatively, non-thermal high-pressure processing at 100 to 600 MPa across two 10 min cycles can lead to leptin retention rates of up to 90% [53]. Ultimately, mother’s own milk is the gold standard for infant feeding, and efforts should be expended to mitigate the disparities that exist in breastfeeding rates and explore alternative methods of processing donated breast milk for optimal delivery of leptin and other trophic or protective factors to exceptionally vulnerable premature infants.

The strengths of our study include the inclusion of infants born at extremely preterm gestations, inclusion of a sufficient number of infants to consider the role of sex as a biologic variable, uniform patient care at a single center, and the use of standardized assessments during follow-up evaluations at the same institution. There are some limitations of our study. While most of the approached families provided consent, 40% of the initial cohort were lost to outpatient follow-up. Given the multifactorial nature of prematurity-related hypertension, we did not exclude infants based on a family history of hypertension or any other risk factor. Because a vast majority of the infants were receiving predominately maternal breast milk by the time they reached 35 weeks’ gestation [24], we were unable to assess the role of different forms of nutrition on the growth or short-term outcomes of the preterm infants. Although we did observe novel relationships between variables of interest and blood pressure or ASQ-3 scores at 2 years, longer-term multicenter follow-up investigations will be necessary to define the longevity and applicability of our observations.

## 5. Conclusions

Consistent with their enhanced risk of neonatal morbidity and mortality, preterm male infants are predisposed to higher blood pressures and lower ASQ-3 scores than their female counterparts. Accounting for that sexual dimorphism and the degree of prematurity, faster postnatal weight gain was associated with higher blood pressures, but independent of growth velocity, higher 35-week PMA leptin levels predicted better performance on the ASQ-3. These findings suggest opportunities for early life nutritional interventions, such as innovatively managed enteral feedings with leptin supplementation used to guide the growth and development of preterm infants towards a goal of optimal long-term neurodevelopment without having to pursue the accelerated growth rates that could disadvantage their cardiovascular outcomes.

## Figures and Tables

**Table 1:** Systolic blood pressure (SBP), diastolic blood pressure (DBP) and total score on the Ages & Stages Questionnaire (ASQ-3) are contrasted for 2-year-old male and female children. Data are presented as median (interquartile range).

	Male	Female	*p*
SBP, mm Hg	99 (92, 107)	95 (90, 104)	0.18
DBP, mm Hg	61 (53, 68)	57 (53, 61)	0.21
ASQ-3, total score	210 (178, 236)	248 (210–265) *	0.003

* *p* < 0.01.

**Table 2:** Systolic blood pressure (SBP, N = 58), diastolic blood pressure (DBP, N = 58) and total score on the Ages & Stages Questionnaire (ASQ-3, N = 66) were correlated by univariate linear regression with maternal and infant variables, including the postnatal leptin levels obtained at 35-week PMA.

		SBP		DBP		ASQ-3	
	Median (IQR)	R	*p*	R	*p*	R	*p*
Gestational age (weeks)	29.6 (27.3, 31.3)	0.30 *	0.02	0.24	0.07	0.24	0.06
Birth weight (g)	1195 (995, 1610)	0.27 *	0.04	0.31 *	0.02	0.20	0.12
Postnatal leptin (pg/mL)	612 (300, 1735)	−0.06	0.68	0.10	0.50	0.19	0.17
Follow-up weight (kg)	11.1 (9.7, 12.7)	0.23	0.09	0.27 *	0.04	−0.01	0.91
Weight gain (g/d)	11.3 (9.7, 13.4)	0.35 *	0.006	0.36 *	0.005	0.10	0.43

* *p* < 0.05.

## Data Availability

The data presented in this study are available on request from the corresponding author. The data are not publicly available to protect patient privacy.
